# Fully Aqueous Electrospinning of Binary PVP/Sodium-Alginate and PVP/Riboflavin Nanofibres: Additive Effects and UV-Assisted Processing

**DOI:** 10.3390/polym18121536

**Published:** 2026-06-20

**Authors:** Julia C. Andrade, Gilmar P. Thim, Fernando Cabral, Frank Jorg Clemens, Marcio Fredel

**Affiliations:** 1 Laboratory for High Performance Ceramics, Empa, Swiss Federal Laboratories for Materials Science and Technology, Überlandstrasse 129, 8600 Dübendorf, Switzerland; frank.clemens@empa.ch; 2 Ceramic and Composite Materials Research Group (CERMAT), Department of Mechanical Engineering, Federal University of Santa Catarina (UFSC), Florianópolis 88040-900, SC, Brazil; fcabral1500@gmail.com (F.C.); mfredelbr@gmail.com (M.F.); 3 Institute of Biomaterials, Department of Materials Science and Engineering, Friedrich-Alexander-Universität Erlangen-Nürnberg (FAU), 91058 Erlangen, Germany; gpthim@gmail.com; 4 Laboratory of Plasmas and Processes, Department of Physics, Instituto Tecnológico de Aeronáutica (ITA), São José dos Campos 12228-900, SP, Brazil

**Keywords:** fully aqueous electrospinning, green electrospinning, PVP-based binary systems, sodium alginate, riboflavin, UV-assisted processing

## Abstract

Electrospinning (ES) can produce nonwoven fibrous mats with high surface area and interconnected porosity, making them attractive for biomedical and functional material applications. However, conventional ES often relies on volatile organic solvents, raising safety, environmental, and translational concerns. Fully aqueous (“green”) ES offers an appealing alternative, although many water-soluble polymers remain difficult to spin and may show limited stability under hydrated conditions. In this study, two fully aqueous binary systems, poly(vinylpyrrolidone)–sodium alginate (PVP–SA) and poly(vinylpyrrolidone)–riboflavin (PVP–RF), were investigated to decouple the roles of sodium alginate (SA) and riboflavin (RF) on solution behaviour, fibre formation, morphology, dry-state mechanical properties, and surface chemistry. Aqueous PVP solutions (20% *w*/*v*; molecular weight 1.3 MDa) were blended with SA (1–5 wt% relative to PVP) or RF (1–10 wt% relative to PVP). Electrical conductivity and rheological properties were evaluated prior to ES under controlled conditions, with simultaneous ultraviolet (UV) exposure at 344 nm during fibre collection. RF did not significantly alter conductivity (~0.74–0.75 µS·cm^−1^), whereas SA increased conductivity up to 2.75 ± 0.03 µS·cm^−1^ at 5 wt%. All formulations exhibited shear-thinning behaviour, while 10 wt% RF increased the zero-shear viscosity relative to neat PVP. Morphological analysis showed that low SA contents produced uniform fibres, whereas higher SA levels (4–5 wt%) led to bead defects and reduced fibre diameter (down to 85 ± 25 nm). Dry-state mechanical performance decreased with increasing SA content, while 10 wt% RF improved tensile strength and toughness, reaching an ultimate tensile strength of 5.21 ± 0.15 MPa and toughness of 40.51 ± 1.53 MJ·m^−3^. Fourier transform infrared spectroscopy (FTIR) and X-ray photoelectron spectroscopy (XPS) indicated subtle UV-driven redistribution of surface chemical states, consistent with mild photo-oxidative microstructural modification rather than extensive covalent network formation. Because the UV irradiance was not directly measured and wet-state stability was not assessed, the UV-related findings are interpreted as preliminary chemical evidence rather than confirmation of stabilized fibre mats. Overall, this work establishes a solvent-free aqueous ES platform in which ionic and photoactive additives can be used to tailor fibre morphology, dry-state mechanical behaviour, and surface characteristics without toxic reagents.

## 1. Introduction

Electrospinning (ES) enables the fabrication of nonwoven fibrous mats with high surface area, interconnected porosity, and tuneable fibre diameters, making it a versatile platform for biomedical, filtration, sensing, and functional material applications [1,2,3,4]. The fibrous architecture can also mimic structural features of native extracellular matrices, which has motivated extensive interest in tissue engineering and drug-delivery systems [5,6]. In addition, the high surface-area-to-volume ratio favours interfacial interactions, rapid wetting, and efficient mass transport, which are valuable across a wide range of membrane-based technologies [7].

However, traditional ES often uses volatile organic solvents such as N-dimethylformamide (DMF) and dimethylacetamide (DMAc), which raise safety, environmental, and translational concerns due to their toxicity and residual contamination. These issues have prompted the development of green ES methods that replace hazardous solvents with safer alternatives while preserving spinnability and fibre uniformity [8,9,10].

Within green ES strategies, water-based processing is particularly attractive because it avoids volatile organic solvents, simplifies post-processing, and can better preserve moisture- or light-sensitive additives compared with conventional solvent systems [11,12]. Fully aqueous ES, therefore, offers a relevant route toward safer and more sustainable fabrication of fibrous materials. However, many water-soluble polymers remain challenging to electrospin because rapid solvent evaporation is absent, jet stability can be reduced, and the resulting fibres often show poor resistance under humid or hydrated conditions [13,14].

Among water-soluble candidates, sodium alginate (SA) is a biodegradable anionic polysaccharide widely used in biomedical, pharmaceutical, and encapsulation technologies [15,16]. From a processing perspective, however, neat SA is difficult to electrospin because its strong polyelectrolyte character promotes charge repulsion between chains, limiting effective molecular entanglement and often resulting in jet instability, bead formation, and discontinuous fibres [17,18].

Blending SA with flexible carrier polymers is a common strategy to enhance spinnability [19,20,21]. While SA blends with poly(vinyl alcohol) (PVA) or poly(ethylene oxide) (PEO) are well documented [22,23]; reports on fully aqueous poly(vinylpyrrolidone) (PVP)–SA systems remain comparatively limited [24].

PVP is an attractive carrier polymer because high-molecular-weight grades provide robust chain entanglement while remaining fully water-soluble [25]. PVP’s lactam groups can form hydrogen bonds with SA hydroxyl and carboxyl moieties, promoting miscibility and fibre cohesion [26,27]. Moreover, PVP is widely used in pharmaceutical and biomedical formulations, supporting its translational relevance [27,28,29].

A key limitation of SA/PVP fibrous mats is their poor aqueous stability, since both polymers are water-soluble [30,31]. Ionic crosslinking of SA is reversible and often unstable in physiological salt solutions due to ion exchange [32,33,34]. This limitation motivates the development of alternative modification strategies that avoid the use of toxic crosslinkers. In this context, ultraviolet (UV)-assisted modification using a photosensitizer has emerged as an efficient and eco-friendly route enabling spatial and temporal control without chemical additives [32,33,34,35].

Among natural photosensitizers, RF, vitamin B2, is especially attractive. It absorbs UV/blue light (370–450 nm) and generates singlet oxygen and radical species via Type I and Type II photochemical pathways [36,37]. RF is clinically applied in corneal collagen crosslinking and demonstrates in vivo safety [38,39]. Unlike synthetic photoinitiators, RF is biocompatible and does not introduce additional toxic residues, making it particularly suitable for green electro-spinning strategies [40].

Importantly, in the present study, we do not fabricate a ternary PVP/SA/RF system. Instead, two binary systems (PVP–SA and PVP–RF) are systematically investigated to decouple the respective roles of SA and RF in modulating solution behaviour, fibre morphology, mechanical response, and surface chemistry. This binary formulation strategy allows SA to be evaluated primarily as an ionic modifier affecting conductivity, jet stability, and fibre formation, while RF is assessed as a photoactive additive for UV-assisted processing with minimal perturbation of solution conductivity.

This study addresses two central challenges: (i) eliminating toxic organic solvents and chemical crosslinkers; (ii) introducing a vitamin-based photoactive additive for UV-assisted processing in a fully aqueous platform. Accordingly, we investigate the effects of SA and RF concentration on solution properties, including electrical conductivity and rheology, as well as ES behaviour, fibre morphology, dry-state mechanical properties, and surface chemistry. Finally, we discuss the role of RF during UV-assisted processing, while acknowledging that direct UV-dose quantification, wet-state stability, and biological validation require dedicated follow-up studies.

## 2. Materials and Methods

PVP (Mw ~1300 kDa), SA (Mw 120–190 kDa), and RF (≥98% purity) were purchased from Sigma-Aldrich (Taufkirchen, Germany) and used as received. Ultrapure water (Milli-Q, 18.2 MΩ·cm, 25 °C) was used as solvent in all preparations.

### 2.1. Preparation of Electrospinning Solutions

A 20% (*w*/*v*) PVP stock solution was prepared by dissolving PVP in ultrapure water under magnetic stirring (C-MAG HS 7, IKA-Werke GmbH & Co. KG, Staufen im Breisgau, Germany) at ~300 rpm and 25 °C for 90 min until complete dissolution. Separately, a 1% (*w*/*v*) SA stock solution was prepared by dispersing SA in ultrapure water using a rotor–stator mixer (Ultra-Turrax T25 Digital, IKA, Staufen, Germany) at 12,000 rpm and 25 °C for 15 min.

Two formulation series were investigated: (i) PVP–SA (SA = 1–5 wt% relative to PVP mass; no RF), stirred for 30 min; (ii) PVP–RF (RF = 1 and 10 wt% relative to PVP mass; no SA). For the PVP–RF series, RF powder was directly added to the aqueous PVP solution and magnetically stirred for 15 min until a visually homogeneous yellow solution/dispersion was obtained. Unless stated otherwise, PVP concentration was maintained at 20% (*w*/*v*) in the final solutions. RF-containing solutions were protected from ambient light during preparation.

Electrical conductivity was measured at 25 °C using a conductometer (912, Metrohm AG, Herisau, Switzerland). Rheological behaviour was analysed at 25 °C using a modular compact rheometer (MCR 302, Anton Paar GmbH, Graz, Austria) after equilibrating each solution for 2 min at rest. Flow curves were recorded using a parallel-plate geometry (25 mm diameter, 1.0 mm gap) with a logarithmic shear-rate ramp from 1 to 1000 s^−1^, collecting ~10 points per decade. Viscosity at each shear rate was taken after torque stabilisation, and no pre-shear conditioning was applied. Each formulation was measured in triplicate to quantify measurement uncertainty.

### 2.2. Electrospinning Procedure and UV Exposure

**Figure 1:**
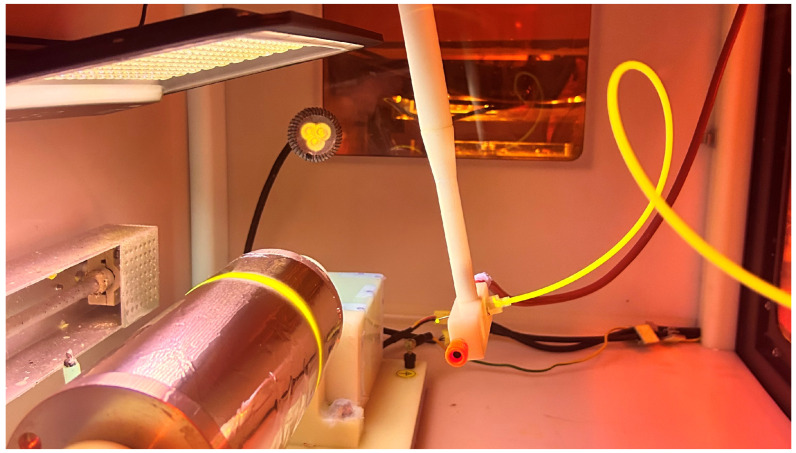
Electrospinning setup for fibre production, showing the syringe–needle system, rotating drum collector, and UV lamp used during the electrospinning procedure.

The ES process (Figure 1) was performed using a NEU-Pro system (NaBond Technologies, Shenzhen, China). Solutions were loaded into 5 mL syringes fitted with 20-gauge stainless steel needles and electrospun at 25 kV, 7 mL·h^−1^, and a 10 cm needle-to-collector distance. Fibres were collected on aluminium foil mounted on a rotating drum collector (diameter 100 mm, length 200 mm, 300 rpm). The chamber temperature was maintained at 80 °C, and the relative humidity at the collector level remained 47–52% (Testo SE & Co. KGaA, Titisee-Neustadt, Germany).

Simultaneously with ES, fibre mats were collected under a 344 nm UV lamp (Spectroline E-Series, Spectronics Corporation, Westbury, NY, USA, 4 W) positioned 10 cm from the collector. The lamp was oriented perpendicular to the rotating drum collector and remained on during the 30 min electrospinning procedure for all samples. Because the actual irradiance at the collector was not available from the manufacturer and was not measured with a radiometer, the total UV dose in J/cm^2^ could not be accurately determined. Therefore, UV exposure is reported in terms of nominal lamp power, wavelength, source-to-collector distance, and exposure time.

Accordingly, UV-related chemical interpretation in the present study was focused specifically on the PVP–RF system, since RF was the photoactive component of interest. The PVP–SA series was primarily used to evaluate the effects of SA content on solution properties, fibre formation, morphology, and mechanical behaviour under the same processing configuration.

### 2.3. Fibre Morphology Characterisation

Fibre morphology was examined by environmental scanning electron microscopy (ESEM; Quanta FEG 650, FEI Company, Hillsboro, OR, USA) which enables imaging of non-conductive, hydrated samples under low-vacuum conditions. Samples were sputter-coated with a 10 nm carbon layer (Leica Microsystems GmbH, Wetzlar, Germany; EM ACE200) and imaged at 5 kV. Fibre diameters were determined with ImageJ from measurements of 100 fibres per sample. Fibre diameters were measured from ESEM micrographs using ImageJ software, version 1.54g (National Institutes of Health, Bethesda, MD, USA). For each formulation, 100 individual fibres were randomly selected from representative images, avoiding beads and fibre intersections. Data are reported as mean ± standard deviation. Statistical analysis was performed using one-way ANOVA followed by Tukey’s post hoc test, with *p* < 0.05 considered statistically significant. For clarity, only comparisons relative to the PVP control are indicated in Table A2.

### 2.4. Mechanical Testing of Fibre Mats

Mechanical properties were measured under uniaxial tension using a universal testing machine (ZwickRoell GmbH & Co. KG, Ulm, Germany; ZwickRoell Z005) equipped with a 200 N load cell. Rectangular specimens (10 mm × 50 mm) were pneumatically clamped (4 bar) and tested at a crosshead speed of 3 mm·min^−1^ with a gauge length of 30 mm. Thickness (0.21 ± 0.05 mm) was measured at five positions per specimen using a digital micrometre and averaged to calculate the initial cross-sectional area.

Ultimate tensile strength (UTS) was calculated as the maximum load divided by the initial cross-sectional area. Toughness was calculated as the area under the stress–strain curve up to failure using trapezoidal numerical integration. Five specimens were tested per condition (*n* = 5). Statistical analysis was performed using one-way ANOVA with Tukey post hoc testing (α = 0.05). Tensile testing was consistent with standard approaches for thin films and nonwoven materials (ASTM D882-18) [41]; ASTM D5035-22 [42]).

### 2.5. Chemical and Surface Characterisation

FTIR spectra were collected by ATR-FTIR (attenuated total reflectance mode) using a Tensor 27 spectrometer (Bruker Optik GmbH, Ettlingen, Germany) in the range 400–4000 cm^−1^, with a spectral resolution of 4 cm^−1^ and 64 scans per sample. FTIR analysis was used to compare PVP and PVP–RF fibre mats before and after UV exposure, with particular focus on identifying UV-associated spectral changes in RF-containing samples rather than directly demonstrating a riboflavin photosensitisation mechanism or covalent network formation.

XPS measurements were performed using a PHI Quantera SXM instrument (ULVAC-PHI Inc., Chigasaki, Japan) equipped with a monochromatic Al Kα source (hν = 1486.6 eV). Survey and high-resolution spectra (C 1s, O 1s, and N 1s regions) were acquired to evaluate surface elemental composition and chemical-state variations before and after UV exposure. The binding-energy scale was calibrated according to ISO 15472:2010 [43]. Data processing and peak fitting were performed using CasaXPS (Casa Software Ltd., Teignmouth, UK; version 2.3.25) with a Shirley background and mixed Gaussian–Lorentzian line shapes. For insulating fibrous mats, charge correction was applied during processing by referencing the C 1s (C–C/C–H) component. Elemental atomic percentages were calculated using the instrument/software sensitivity factors.

## 3. Results and Discussion

### 3.1. Electrical Conductivity of Electrospinning Solutions

Electrical conductivity is a key electrohydrodynamic parameter in ES because it governs the amount of charge carried by the jet. Increasing conductivity generally increases charge density, which enhances electrostatic stretching and bending (whipping) instabilities, favouring jet thinning and often smaller fibre diameters [44]. However, when the charge density exceeds the solution’s viscoelastic resistance, jet stability can deteriorate, leading to defects such as beads and diameter fluctuations. Conductivity should therefore be interpreted in conjunction with rheology and the resulting fibre morphology [40,44].

**Figure 2:**
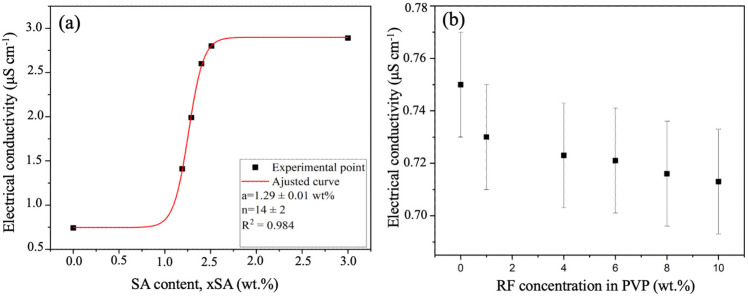
Electrical conductivity of aqueous electrospinning solutions. (**a**) Effect of sodium alginate (SA) content on conductivity; symbols represent experimental data, and the solid line is the fitted saturation model. (**b**) Effect of riboflavin (RF) content on conductivity; symbols represent experimental data (mean ± SD). Experiments in triplicate (*n* = 3).

Figure 2 summarises the conductivity of fully aqueous PVP-based formulations as a function of SA and RF contents. Neat PVP exhibits low conductivity, consistent with its non-ionic character and the limited population of mobile charge carriers in water. Importantly, incorporating RF (0–10 wt% relative to PVP) does not increase conductivity beyond the experimental uncertainty.

Across the tested range, values remain close to the PVP control, indicating that RF does not introduce a significant concentration of mobile ions under the present conditions. The small decrease observed at higher RF contents is comparable to the error bars and is therefore not interpreted as a systematic effect. This limited effect is likely related to the relatively low RF concentration and to the fact that, under the present conditions, RF does not behave as a strong electrolyte or polyelectrolyte in solution. Practically, these results indicate that RF can be incorporated as a photoactive additive without significantly affecting solution conductivity [40].

In contrast, adding SA markedly increases conductivity, reflecting its polyelectrolyte nature. Upon dissolution, SA dissociation and the release of ions contribute to charge transport, increasing the solution’s ability to carry current [45]. The increase is strongly non-linear: conductivity rises steeply at low SA contents and then approaches an upper limit close to the conductivity of the SA-rich control. Such saturating behaviour is consistent with ionic-strength effects, ion association, and reduced charge mobility at higher polyelectrolyte contents, which can reduce the incremental contribution of additional SA to the pool of effectively mobile ions [46,47]. A Hill-type saturation model conveniently describes the SA-dependent data [48]:

(1) $$S\left(x_{SA}\right)=\;S_{SA}-\frac{S_{PVP\;}-S_{SA\;}}{1+{\left.\left(\frac{x_{SA}}{a}\right.\right)}^{n}}$$

where S is the electrical conductivity (μS cm^−1^), X_SA_ is the SA content (wt%), S_PVP_ is the conductivity of the neat PVP control solution, S_SA_ is the conductivity of the neat SA control solution, and *a* and *n* are fitted parameters. Parameter *a* represents the characteristic SA content at which the transition occurs (half-saturation when *n* is large), while *n* controls the steepness of the rise.

Using the current dataset, the fitted parameters were a = 1.29 ± 0.01 wt% and n = 14 ± 2 (R2 = 0.984). The large *n* indicates a sharp transition in conductivity over a narrow SA range, consistent with the rapid rise observed experimentally at low SA contents [47]. Although the model is empirical, it provides a compact description of the data and can be used to interpolate conductivity within the tested formulation window.

From an ES standpoint, these trends highlight distinct roles for SA and RF in fully aqueous PVP solutions. SA acts primarily as an electrical modifier, increasing conductivity and thereby intensifying electrostatic stretching, which is expected to favour smaller fibre diameters [49]. At the same time, higher charge density can intensify capillary and bending instabilities and thus increase the risk of bead formation if jet viscoelasticity is insufficient, especially at higher SA contents [47].

RF, in contrast, leaves conductivity essentially unchanged and therefore is unlikely to introduce conductivity-driven instabilities; any effects on fibre formation should instead arise from changes in viscosity and intermolecular interactions. Taken together, the conductivity results support the interpretation that SA governs the electrohydrodynamic driving force for jet thinning. In contrast, RF can be incorporated under the UV-assisted processing conditions used here with minimal perturbation of electrical transport.

### 3.2. Rheological Behaviour of Electrospinning Solutions

**Figure 3:**
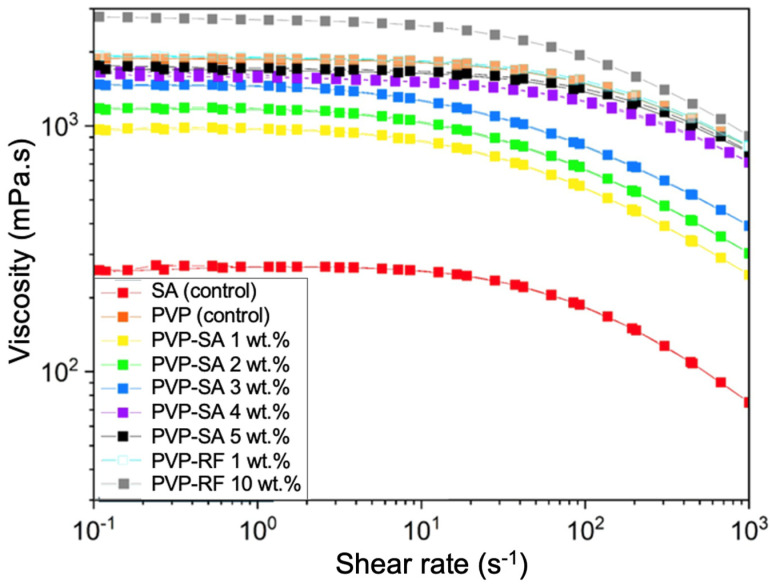
Flow curves of aqueous poly(vinylpyrrolidone) (PVP), PVP–SA, and PVP–RF solutions at different compositions, showing shear-thinning behaviour over a wide viscosity range; measurements were performed in triplicate (*n* = 3). Symbols represent the average experimental measurements; lines are included only to guide the eye.

All PVP-based formulations exhibited non-Newtonian shear-thinning behaviour (Figure 3), which is typical of ES solutions and generally beneficial for processing. Under the high shear and extensional stresses experienced near the needle and along the electrified jet, a progressive decrease in viscosity facilitates jet elongation and thinning [50,51].

At the same time, a sufficiently high low-shear viscosity is required to maintain chain entanglement and suppress jet breakup, which is a key prerequisite for bead-free fibre formation [52]. Because electrostatic stretching increases with conductivity (Section 3.1), rheology and conductivity should be interpreted together: the balance between electrical forcing and viscoelastic stabilisation sets jet stability [53].

At low shear rates, the solutions approached a quasi-Newtonian plateau that defines the zero-shear viscosity (η0). Above a characteristic shear rate, viscosity decreased markedly, indicating the onset of shear-thinning. The flow curves were fitted using the Cross model [54]:

(2) $$\eta (\dot{\gamma })=\;\eta \infty \;+\;\frac{\eta 0-\;\eta \infty }{1+{\left(\tau \cdot \dot{\gamma }\right)}^{1-n}}$$

where η0 is the zero-shear viscosity, η∞ is the infinite-shear viscosity (assumed negligible here), $\dot{\gamma }$ is the shear rate, τ is a characteristic time constant, and *n* is the shear-thinning exponent (0 < n < 1). Within this framework, the onset of shear-thinning can be estimated as the critical shear rate γc ≈ 1/τ, providing a compact metric for comparing formulations (Table A1).

Neat PVP showed η0 = 1898 ± 5 mPa·s and a transition to shear-thinning at γc ≈ 662 s^−1^. Incorporation of low SA contents (1–2 wt%) produced only modest reductions in η0 (1742–1648 mPa·s) and similar γc values (676–752 s^−1^), indicating that small SA additions do not strongly disrupt the viscoelastic window required for stable spinning. In contrast, higher SA contents (≥3 wt%) progressively reduced η0 down to 1006 ± 6 mPa·s and shifted the onset of shear-thinning to lower shear rates (γc ≈ 146–153 s^−1^).

This combination of lower low-shear viscosity and higher conductivity (Section 3.1) is expected to increase jet instabilities when chain entanglement becomes insufficient, which is consistent with the bead-rich morphologies observed at higher SA contents (Section 3.3) and the deterioration in mat mechanical performance (Section 3.4). A practical contributor to the decrease in viscosity at higher SA loadings may be the partial dilution of the PVP matrix when SA is introduced from an aqueous stock solution.

RF affected rheology differently. At 1 wt% RF, the flow curve nearly overlapped with neat PVP (η0 = 1929 ± 3 mPa·s; γc ≈ 645 s^−1^), indicating that low RF loading had little effect on the measured flow behaviour. In contrast, 10 wt% RF increased η0 to 2777 ± 6 mPa·s and shifted the onset of shear-thinning to lower shear rates (γc ≈ 326 s^−1^). This result indicates that RF affected the rheological response only at the higher loading investigated. Since no particle-size, solubility, or molecular-interaction analysis was performed, the origin of this viscosity increase cannot be assigned unambiguously. Nevertheless, interactions involving RF and polymeric matrices have been reported in related systems. Importantly, this viscosity increase occurs without a meaningful increase in conductivity (Section 3.1), helping to preserve stable jet formation while supporting viscoelastic stabilisation.

The rheological results support distinct formulation roles: SA primarily introduces ionic character and raises conductivity while progressively narrowing the viscoelastic stabilisation window at higher contents, whereas RF affected the rheological response mainly at the higher loading investigated, without perturbing conductivity. This decoupling is advantageous for fully aqueous electrospinning, as it enables the incorporation of RF as a photoactive additive without significantly altering electrohydrodynamic conditions [55,56].

### 3.3. Fibre Morphology

**Figure 4:**
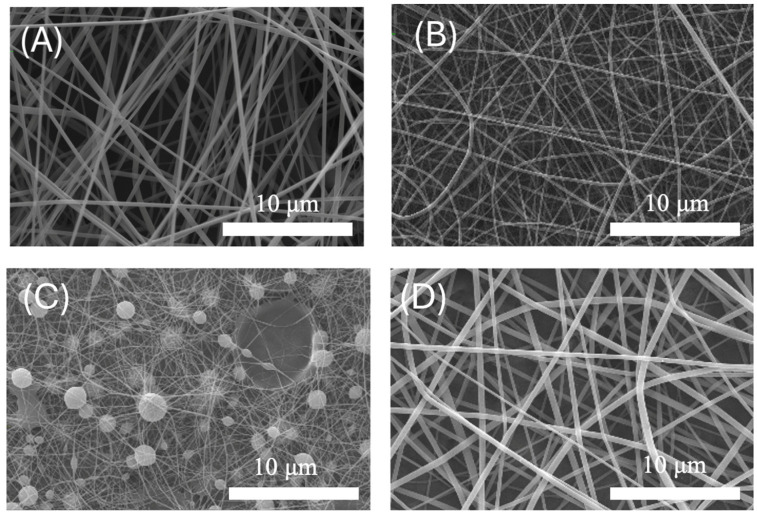
Representative ESEM micrographs of electrospun mats from PVP, PVP–RF and PVP–SA formulations, highlighting the transition to bead-like defects at high SA contents and the defect-free morphology of RF-containing fibres. (**A**) PVP pure; (**B**) PVP-SA 3 wt% SA; (**C**) PVP-SA 5 wt%; (**D**) PVP-RF 10 wt%.

ESEM was used to assess the morphology of electrospun fibres. Figure 4 shows representative micrographs selected to highlight the main morphological differences among formulations, while the full-diameter analysis for all groups is provided in Table A2. The macroscopic appearance of the corresponding fibre mats is presented in Supplementary Figure S1. The results show that composition strongly governs fibre uniformity and diameter in this fully aqueous system. These trends align with the coupled changes in electrical conductivity (Section 3.1) and viscoelastic response (Section 3.2), which together control (i) electrostatic stretching of the jet and (ii) the ability of the filament to resist capillary breakup during flight [57].

Neat PVP produced continuous, smooth, and bead-free fibres. Introducing SA at low contents (1–3 wt%) preserved fibre continuity and surface smoothness, indicating that the PVP matrix still provides sufficient chain entanglement for stable spinning. At higher SA contents (4–5 wt%), the morphology shifted towards thinner, less uniform fibres with frequent bead-like defects (a bead-on-string appearance).

This loss of uniformity is consistent with the sharp increase in solution conductivity induced by SA (Section 3.1), which increases the jet charge density and amplifies electrostatic stretching and whipping [56]. When these stronger electrohydrodynamic forces are not matched by adequate viscoelastic stabilisation, capillary instabilities become more prominent, favouring bead formation and diameter fluctuations [58]. Notably, the SA-rich formulations also exhibit reduced zero-shear viscosity relative to neat PVP (Section 3.2), suggesting that the combination of higher charge density and a weaker viscoelastic window provides a plausible mechanistic basis for the observed defects.

In contrast, adding RF to PVP (1–10 wt%) did not compromise fibre uniformity. Both RF-containing formulations yielded smooth, defect-free fibres, supporting the conclusion from Section 3.1 that RF does not materially alter solution conductivity and therefore does not introduce conductivity-driven instabilities. At the same time, rheology indicates that 10 wt% RF increases viscosity (Section 3.2), which can improve jet cohesion and suppress bead-forming breakup, while also reducing the extent of jet thinning. Thus, RF can be incorporated for UV exposure while maintaining relatively constant electrohydrodynamic conditions and, at higher loading, potentially improving spinnability via viscosity-mediated jet stabilisation.

Quantitative diameter analysis (Table A2) corroborates the ESEM observations. PVP fibres exhibited an average diameter of 128 ± 31 nm. RF at 1 wt% produced a comparable diameter (132 ± 28 nm), whereas 10 wt% RF increased the diameter to 183 ± 35 nm, consistent with reduced jet thinning at higher viscosity. By contrast, increasing SA content progressively decreased the diameter from 117 ± 20 nm (1 wt% SA) to 85 ± 25 nm (5 wt% SA), consistent with greater electrostatic stretching in the more conductive, SA-rich solutions.

However, at high SA contents, the benefit of a smaller average diameter is offset by the concurrent rise in bead defects and non-uniformity. Thus, the morphology data reinforce distinct formulation roles of the additives. SA primarily acts as an electrical modifier, promoting jet stretching and thinner fibres, but can drive bead formation when conductivity increases while viscoelastic stabilisation diminishes.

RF, in contrast, can be incorporated as a photoactive functional additive under the UV-assisted processing conditions used here, with minimal perturbation of solution conductivity; at higher loading, it increases viscosity, yielding thicker, yet uniform, fibres. These morphology trends anticipate the mechanical results (Section 3.4), where bead-rich mats are expected to exhibit reduced strength and toughness due to stress concentrators and poorer load transfer across the fibrous network.

### 3.4. Mechanical Properties

**Figure 5:**
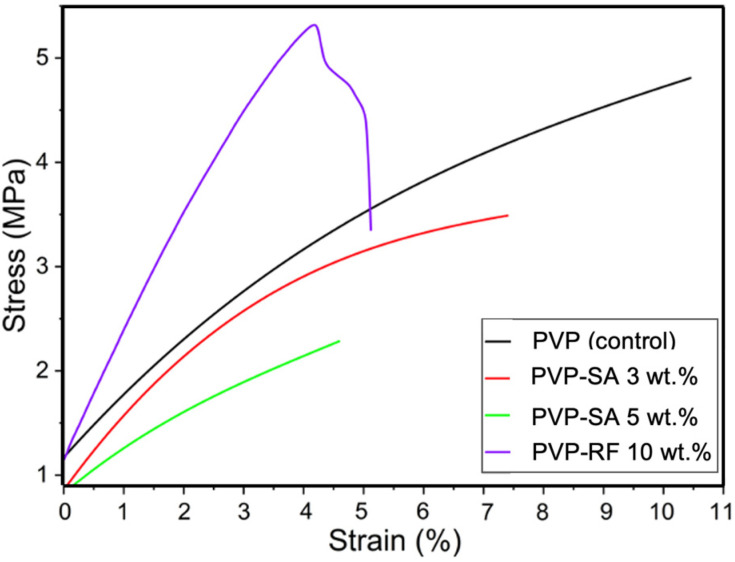
Stress–strain curves of mechanically tested electrospun PVP, PVP–SA, and PVP–RF fibre mats. PVP–RF 10 wt% showed the highest strength and toughness, whereas increasing SA content (3–5 wt%) reduced mechanical performance.

Uniaxial tensile testing (Figure 5) was used to evaluate the mechanical integrity of selected electrospun mats. All mechanically tested groups were evaluated under identical conditions (n = 5; same specimen geometry and strain rate). The responses were quantified as ultimate tensile strength (UTS) and toughness (area under the stress–strain curve), which together describe the load-bearing capacity and energy absorption of the non-woven mats. Although electrospun mats may exhibit intrinsic heterogeneity, the dispersion observed in the present measurements was low, and the main comparative trends were larger than the within-group scatter [59].

Neat PVP mats showed an intermediate mechanical performance (UTS = 4.67 ± 0.10 MPa; toughness = 35.08 ± 1.23 MJ m^−3^). Adding SA progressively reduced both metrics. At 3 wt% SA, UTS decreased to 3.66 ± 0.29 MPa and toughness to 19.23 ± 1.27 MJ m^−3^, while at 5 wt% SA the mats dropped further to 2.40 ± 0.42 MPa and 9.73 ± 2.16 MJ m^−3^, respectively. One-way ANOVA confirmed a significant composition effect for both UTS (*p* = 0.005) and toughness (*p* < 0.0001).

This deterioration is consistent with the microstructural changes reported in Section 3.3: at higher SA contents, the fibres become thinner and increasingly bead-rich, and beads act as stress concentrators, reducing effective load transfer across the network. In addition, SA simultaneously increases conductivity (Section 3.1) while decreasing viscosity at higher loadings (Section 3.2), thereby amplifying electrohydrodynamic instabilities and further promoting defects and heterogeneity, ultimately compromising mat mechanics.

RF affected mechanics in the opposite direction. PVP–RF 10 wt% mats reached UTS = 5.21 ± 0.15 MPa and toughness = 40.51 ± 1.53 MJ m^−3^, corresponding to ~12% higher strength and ~15% higher toughness than neat PVP. Two coupled factors are likely to contribute to this reinforcement. First, at high loading, RF increased the zero-shear viscosity (Section 3.2) without materially changing conductivity (Section 3.1), favouring stable jet formation and producing smooth, bead-free fibres (Section 3.3). Second, RF may introduce additional non-covalent interactions in the solid state, such as hydrogen bonding with PVP carbonyl/lactam groups and/or RF self-association, which could act as supramolecular constraints within the fibre matrix. Because all mats were collected under the same UV exposure conditions, the improved performance of PVP–RF 10 wt% mats is interpreted primarily in relation to RF incorporation and associated rheological/morphological changes, while any contribution from UV-associated effects remains indirect and should be interpreted cautiously [60,61].

For instance, the dry-state mechanical results provide a comparative indication of mat integrity under the present testing conditions and can be discussed within the broader context of mechanically relevant electrospun fibrous materials [62]. The opposing effects of SA and RF highlight a central formulation trade-off in fully aqueous ES: increasing ionic conductivity can help electrostatic stretching and reduce fibre diameter, but it can also intensify jet instabilities and introduce defects unless counterbalanced by sufficient viscoelastic stabilisation and cohesive interactions. Future work should complement these dry-state tests with wet-state mechanical measurements, swelling, dissolution, gel-fraction analysis, and longer-term incubation in physiologically relevant media to determine whether the electrospun mats retain sufficient integrity under hydrated conditions [63].

### 3.5. Structural Changes Associated with UV Irradiation

**Figure 6:**
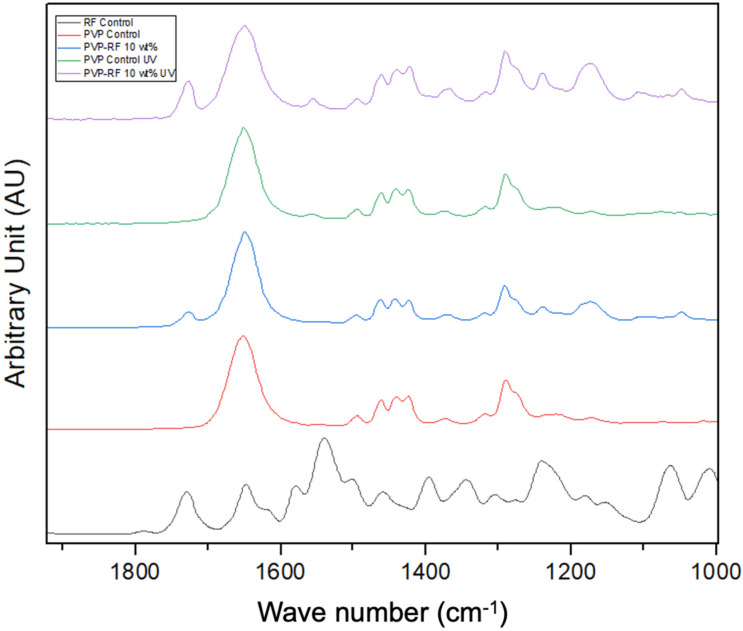
FTIR spectra of RF control, PVP control, PVP–RF 10 wt% fibres, UV-treated PVP fibres, and UV-treated PVP–RF 10 wt% fibres in the 1900–1000 cm^−1^ region. The spectra highlight the dominant PVP lactam C=O band near ~1650 cm^−1^ and RF/PVP-related contributions in the ~1650–1570 cm^−1^ and 1300–1400 cm^−1^ regions. UV-treated PVP–RF fibres show only modest changes in band intensity and shape, without the appearance of distinct new bands indicative of covalent PVP–RF network formation.

FTIR analysis was performed to evaluate possible UV-associated spectral changes in PVP and PVP–RF fibre mats before and after UV exposure (Figure 6). Accordingly, the UV-related chemical interpretation in this section is intentionally restricted to spectral changes observed in the PVP–RF formulations, in which riboflavin was incorporated as the photoactive component of interest, rather than to directly demonstrate a riboflavin photosensitisation mechanism or covalent crosslink formation. In the non-irradiated PVP–RF 10 wt% sample, the strong band near ~1650 cm^−1^ is mainly assigned to the PVP lactam C=O stretching vibration, with possible overlap from RF-related contributions. After UV treatment, only subtle changes are observed in the relative intensity and shape of the ~1650–1570 cm^−1^ region, as well as in the 1300–1400 cm^−1^ region, indicating a modified local chemical environment rather than the formation of a distinctly new network structure [40].

Importantly, UV exposure does not generate new FTIR bands that would provide direct evidence of covalent bond formation between PVP and riboflavin. Instead, the spectral differences are limited to modest variations in band intensity and position. Bands between 1650 and 1570 cm^−1^, associated with C=O and C=N vibrations, are more pronounced in RF-containing fibres, consistent with RF-related contributions and possible changes in intermolecular interactions within the fibre matrix [64]. 

In addition, a weak feature around ~1550 cm^−1^ is observed in UV-treated samples, which may be associated with subtle changes induced by irradiation, although the low intensity and overlap with neighbouring bands prevent definitive assignment. Additional variations are observed in the 1300–1400 cm^−1^ region, characteristic of C–N stretching vibrations, where a previously weak band becomes more intense and is slightly shifted after UV treatment; such changes are compatible with mild UV-associated chemical micro-adjustments and/or changes in non-covalent interactions within the PVP–RF fibre matrix, but they do not demonstrate the formation of a new covalent PVP–RF network [65].

Therefore, the FTIR results indicate subtle UV-induced local structural changes, without direct evidence of improved wet stability or covalent crosslinking. In this context, the observed spectroscopic changes are compatible with UV-associated modifications in fibres containing riboflavin, suggesting limited photo-oxidative effects while preserving the overall molecular character of the PVP-based fibre matrix [66]. Because riboflavin is photoactive, dedicated UV–Vis absorption and/or fluorescence spectroscopy of RF-containing solutions, fibre extracts, or irradiated RF controls would be required to directly assess RF retention, photobleaching, or possible photodegradation after UV exposure. Such analyses were not performed in the present study.

Accordingly, the present FTIR results should be interpreted as evidence of limited UV-associated changes in the PVP–RF fibre mats, rather than as direct proof of RF photochemical stability, photosensitisation efficiency, wet-state stabilisation, or extensive covalent crosslinking.

### 3.6. Surface Chemistry and UV-Induced Modifications

**Figure 7:**
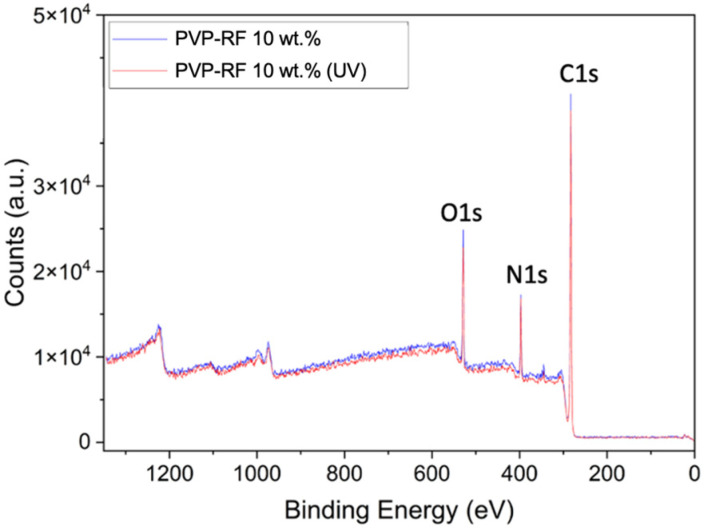
XPS survey spectra of PVP–RF 10 wt% fibres before and after UV treatment. The spectra show the characteristic C 1s, O 1s, and N 1s signals expected for PVP-based fibres. The strong overlap between the profiles indicates no measurable change in overall surface elemental composition within the XPS detection limits.

XPS was used to examine the surface chemical composition of PVP–RF fibres before and after UV exposure. The survey spectra (Figure 7) showed the expected C 1s (~285 eV), O 1s (~532 eV), and N 1s (~400 eV) signals for PVP-based materials, with no additional elements detected. Because XPS probes only the outermost few nanometres of the fibre surface, it is particularly sensitive to surface chemical-state variations rather than bulk compositional changes. The strong overlap of the survey spectra indicates that UV treatment did not produce a measurable change in the overall surface elemental composition within the technique’s detection limits.

**Figure 8:**
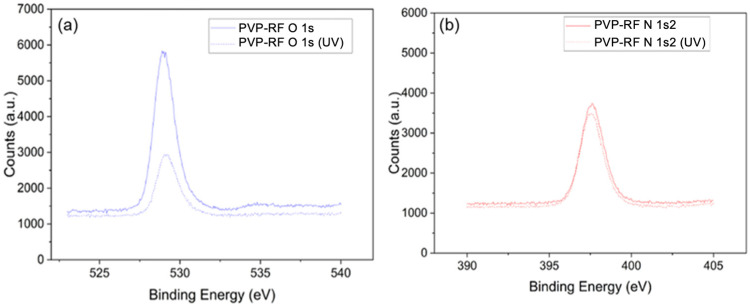
High-resolution XPS spectra of PVP–RF 10 wt% fibres before and after UV treatment. (**a**) O 1s high-resolution spectrum showing slight variations in peak shape after UV exposure; (**b**) N 1s high-resolution spectrum showing minimal changes following UV treatment. Overall, only subtle changes in chemical-state distribution are observed after irradiation, consistent with minor surface-level chemical-state rearrangements rather than definitive evidence of extensive covalent crosslink formation, wet-state stabilisation, or a complete RF-mediated photosensitisation mechanism.

High-resolution analysis of the O 1s and N 1s regions (Figure 8) revealed only subtle spectral changes after UV irradiation. In the O 1s region, a small variation in peak shape and/or binding-energy distribution was observed, suggesting a limited redistribution of oxygen-containing surface chemical states, such as changes in the relative contributions of C=O and C–O environments. In the N 1s region, the peak position and line shape remained largely unchanged, indicating that the nitrogen environment was only minimally affected by UV exposure. The C 1s signal also remained dominated by the polymer backbone, with no pronounced new contributions indicative of a major chemical transformation at the surface.

These results are consistent with mild UV-induced surface oxidation or local chemical-state rearrangements, without evidence of extensive covalent crosslinking [67] In this sense, the XPS data should be interpreted as evidence of subtle surface-level chemical modifications, rather than as direct proof of improved wet integrity, bulk stabilisation, riboflavin-mediated photosensitisation efficiency, or crosslink formation. This interpretation is in line with previous reports in the literature, in which small changes in O 1s and C 1s components after UV exposure were associated with photo-oxidative surface effects and local rearrangements in polymeric systems [68,69].

The XPS results should be interpreted as a complement the FTIR analysis by indicating that UV exposure in RF-containing fibres results in only limited surface chemical changes. Together, these observations support a cautious interpretation of mild UV-associated modification in the PVP–RF system, possibly involving local chemical-state rearrangements and changes in non-covalent interactions, without demonstrating the formation of a new covalent network [70]. Therefore, the XPS data should not be interpreted as direct evidence of wet-state stabilisation or as proof of a complete RF photosensitisation mechanism.

## 4. Conclusions

This study demonstrates a fully aqueous electrospinning (ES) strategy based on two binary systems, PVP–SA and PVP–RF, allowing the respective effects of ionic and photoactive additives to be systematically distinguished within a solvent-free platform.

SA primarily acted as an electrohydrodynamic modifier, significantly increasing solution conductivity and promoting stronger jet stretching, which reduced fibre diameter. However, at higher SA contents (≥4 wt%), the simultaneous decrease in zero-shear viscosity narrowed the viscoelastic stabilisation window, leading to bead formation and reduced mechanical performance. These results highlight the need to balance charge density and chain entanglement when designing fully aqueous ES formulations.

In contrast, RF preserved the low-conductivity character of PVP solutions while increasing viscosity at higher loading (10 wt%), supporting stable jet formation and uniform fibre morphology. RF-containing mats also showed improved tensile strength and toughness relative to neat PVP, indicating that RF might contribute as a photoactive additive under the UV-assisted processing conditions used here and as a possible supramolecular reinforcing component within the fibrous network. However, the present data do not directly demonstrate the complete RF photosensitisation mechanism.

FTIR and XPS analyses revealed only subtle UV-associated spectral and surface chemical-state changes, without evidence of extensive covalent crosslinking. These findings are compatible with mild UV-associated microstructural adjustments and possible changes in non-covalent interactions, rather than permanent covalent network formation. Therefore, the spectroscopic results should not be interpreted as direct proof of wet-state stabilisation, RF photochemical stability, or extensive RF-mediated crosslinking.

Finally, this work establishes a green ES route that eliminates toxic organic solvents and avoids conventional chemical crosslinkers while enabling controlled modulation of fibre morphology, mechanical behaviour, and surface characteristics. The binary formulation approach provides a practical framework for designing fully aqueous nanofibrous materials for biomedical and other functional applications, while also helping to clarify the distinct roles of SA and RF separately, before more complex ternary formulations are considered.

Future studies should examine the effects of measured UV irradiance, total UV dose, and exposure time, as well as RF retention and possible photodegradation using UV–Vis absorption and/or fluorescence spectroscopy. In addition, wet stability, swelling, dissolution, gel fraction, wet-state mechanical behaviour, and long-term stability under relevant hydrated conditions should be evaluated before biomedical translation. After the most promising formulations have been selected and their hydrated stability confirmed, biological validation should include in vitro cytocompatibility, cell adhesion, proliferation, and degradation-related assays. Depending on the intended application, later-stage in vivo studies may then be considered.

## Data Availability

The data presented in this study are available within the article and Supplementary Materials. Further inquiries can be directed to the corresponding author.

## Supplementary Materials

The following supporting information can be downloaded at: https://www.mdpi.com/article/10.3390/polym18121536/s1, Figure S1: Photograph of the complete set of electrospun nanofiber mats corresponding to the formulations investigated in this study.

## Appendix A

**Table A1:** Cross-model fit parameters for the shear viscosity of ES solutions. Critical shear rate γc was estimated as 1/τ from the Cross-model time constant τ. Values are reported as mean ± SD from the model fits.

Solution	Zero Shear Viscosity η0 (mPa·s)	Cross Exponent “*n*”	Critical Shear Rate, γc (s^−1^)
SA (control)	270 ± 2	0.19 ± 0.02	271.0
PVP-SA 1 wt%	1742 ± 3	0.28 ± 0.01	751.9
PVP-SA 2 wt%	1648 ± 7	0.40 ± 0.02	675.7
PVP-SA 3 wt%	1524 ± 9	0.41 ± 0.01	145.7
PVP-SA 4 wt%	1221 ± 7	0.38 ± 0.01	146.6
PVP-SA 5 wt%	1006 ± 6	0.35 ± 0.02	153.4
PVP (control)	1898 ± 5	0.24 ± 0.01	662.3
PVP-RF 1 wt%	1929 ± 3	0.28 ± 0.01	645.2
PVP-RF 10 wt%	2777 ± 6	0.31 ± 0.01	325.7

**Table A2:** Fibre diameter of PVP, PVP–RF, and PVP–SA electrospun fibres. Data are presented as mean ± SD from 100 fibre measurements per formulation. Statistical differences were assessed by one-way ANOVA followed by Tukey’s post hoc test. For clarity, only comparisons relative to the PVP control are indicated. The dash “–” indicates the PVP reference group,.values marked with “*” are significantly different from the PVP control (*p* < 0.05), whereas “ns” indicates no statistically significant difference (*p* ≥ 0.05).

Fibre Composition	Average Fibre Diameter (nm)	Statistical Difference
PVP (control)	128 ± 31	–
PVP-RF 1 wt%	132 ± 28	ns
PVP-RF 10 wt%	183 ± 35	*
PVP-SA 1 wt%	117 ± 20	*
PVP-SA 2 wt%	109 ± 33	*
PVP-SA 3 wt%	105 ± 19	*
PVP-SA 4 wt%	92 ± 41	*
PVP-SA 5 wt%	85 ± 25	*
