# Supplementary material for: Fully Aqueous Electrospinning of Binary PVP/Sodium-Alginate and PVP/Riboflavin Nanofibres: Additive Effects and UV-Assisted Processing
Source: Polymers (Basel). 2026 Jun 20;18(12):1536. doi: 10.3390/polym18121536 (PMC13306411; doi:10.3390/polym18121536)

Supplementary Figure S1. Photograph of the complete set of electrospun nanofiber mats corresponding to the formulations investigated in this study. Supplementary Figure S1. Macroscopic photograph of the electrospun nanofibre mats investigated in this study. Samples are shown from left to right, top row first, then bottom row: S1 = PVP, S2 = PVP–RF 1 wt.%, S3 = PVP–RF 10 wt.%, S4 = PVP–SA 1 wt.%, S5 = PVP–SA 2 wt.%, S6 = PVP–SA 3 wt.%, S7 = PVP–SA 4 wt.%, and S8 = PVP–SA 5 wt.%. Scale bar: 1 cm.

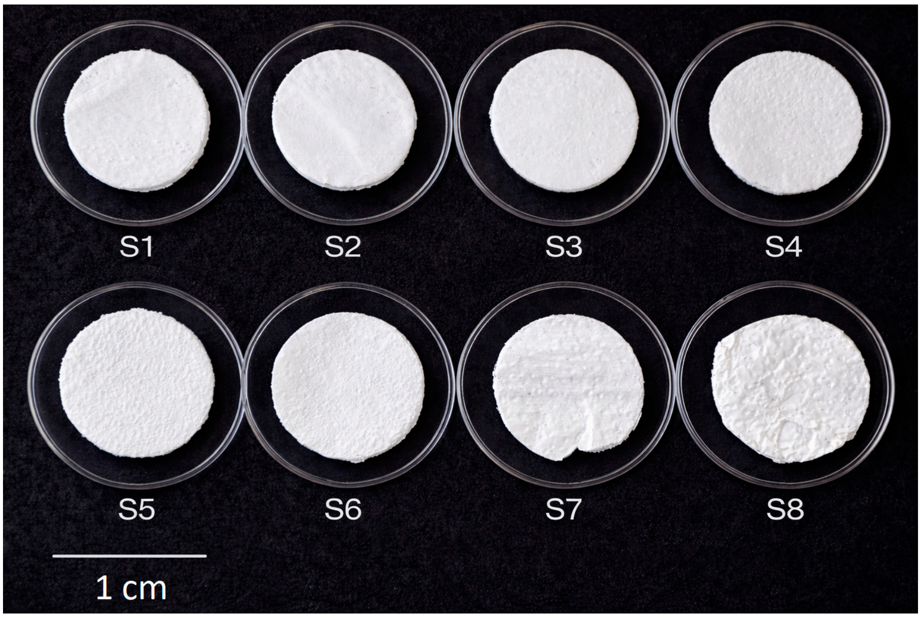

Supplement: Supplementary file 1 [file polymers-18-01536-s001.zip › polymers-4351121-supplementary.pdf]
